# Nomogram for Predicting Survival in Advanced Gastric Cancer after Neoadjuvant Chemotherapy and Radical Surgery

**DOI:** 10.1155/2021/2923700

**Published:** 2021-07-28

**Authors:** Yonghe Chen, Dan Liu, Jian Xiao, Jun Xiang, Aihong Liu, Shi Chen, Junjie Liu, Xiansheng Hu, Junsheng Peng

**Affiliations:** ^1^Department of Gastric Surgery, The Sixth Affiliated Hospital, Sun Yat-sen University, Guangzhou 510655, China; ^2^Guangdong Institute of Gastroenterology, Guangdong Provincial Key Laboratory of Colorectal and Pelvic Floor Diseases, Guangzhou 510655, China; ^3^Department of Laboratory Science, The Second Affiliated Hospital of Guangzhou University of Chinese Medicine, Guangzhou 510105, China; ^4^Department of Medical Oncology, The Sixth Affiliated Hospital, Sun Yat-sen University, Guangzhou 510655, China

## Abstract

**Background:**

Neoadjuvant chemotherapy (NAC) with subsequent radical surgery has become a popular treatment modality for advanced gastric cancer (AGC) worldwide. However, the survival benefit is still controversial, and prognostic factors remain undetermined.

**Aim:**

To identify clinical parameters that are associated with the survival of AGC patients after NAC and radical surgery and to establish a nomogram integrating multiple factors to predict survival.

**Methods:**

We reviewed the medical profiles of 215 AGC patients who received NAC and radical resection, and clinical parameters concerning NAC, surgery, pathological findings, and adjuvant chemotherapy were analyzed using a Cox regression model to determine their impact on survival. Based on these factors, a nomogram was developed and validated.

**Results:**

The overall 1-year and 3-year survival rates were 85.8% and 55.6%, respectively. Younger age (<60 years old), increased examined lymph nodes (exLNs), successful R0 resection, the achievement of pathological complete response (pCR), and acceptance of adjuvant chemotherapy were positive predictors of survival. The C-index of the established nomogram was 0.785. The area under receiver operating curve (ROC) at 1/3 years of prediction was 0.694/0.736, respectively. The model showed an ideal calibration following internal bootstrap validation.

**Conclusion:**

A nomogram predicting survival after NAC and surgery was established. Since this nomogram exhibited satisfactory and stable predictive power, it can be inferred that this is a practical tool for predicting AGC patient survival after NAC and radical surgery.

## 1. Introduction

Gastric cancer is the fifth most common malignancy in the world and the third leading cause of cancer-related death [1]. The majority of affected patients are diagnosed at an advanced stage due to the insidious onset of this malignancy, especially in China [2], resulting in poor survival. In recent years, neoadjuvant chemotherapy (NAC) was introduced into the integrated treatment modality for advanced gastric cancer (AGC) and soon became popular [3]. Some scholars stated that NAC could result in tumor downstaging and a higher curative resection rate and may eventually prolong survival for AGC patients [4–9]. However, some other trials failed to prove any survival benefit from NAC [10–12]. Moreover, well-designed prospective trials are still lacking. Thus, the necessity of NAC for AGC patients is still controversial [13].

To achieve precision medication, the decision of whether to administer NAC should be determined according to the potential survival gain [14]. Thus, it is important to address the survival predictors for patients who receive NAC and surgery. A few studies set out to identify the factors that predict survival after NAC [15–19]. However, these studies only addressed the impact of singular factors without systematically studying all possible factors or combining them. A practical model integrating multiple factors to predict survival after NAC and surgery is still lacking.

Therefore, the purpose of this study was to identify clinical parameters that have predictive value on survival and their weights of impact. Second, we aimed to establish a nomogram integrating multiple factors to predict survival in an attempt to offer a larger picture of survival after the combined treatment modality of NAC with subsequent radical surgery and to provide a practical tool for clinical use.

## 2. Materials and Methods

### 2.1. Study Population and Data Collection

In the initial screening process, we identified 1346 patients who underwent radical gastrectomy from the gastric cancer database of The Sixth Affiliated Hospital, Sun Yat-sen University, from March 2012 to December 2019.

**Figure 1:**
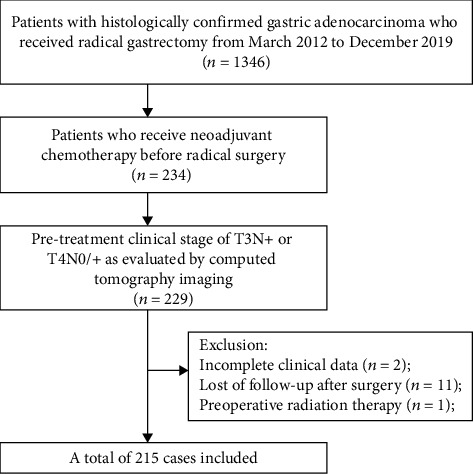
Flow chart of the inclusion and exclusion process.

The inclusion criteria were as follows: (i) histologically confirmed adenocarcinoma of the stomach or esophagogastric junction; (ii) a clinical stage of T3N+ or T4N0/+ as evaluated by computed tomography imaging; (iii) administration of NAC followed by gastrectomy with standardized D2 lymphadenectomy. The exclusion criterion was as follows: (i) incomplete clinical data; (ii) lost follow-up after surgery; (iii) preoperative radiation therapy. The inclusion and exclusion process is depicted in Figure 1.

Finally, a total of 215 patients were included in this study. Information on demographics, including sex, age, body mass index (BMI), biopsy pathological tumor grade, and clinical stage, was collected.

This retrospective study was approved by the Institutional Review Board of The Sixth Affiliated Hospital, Sun Yat-sen University.

### 2.2. Neoadjuvant Chemotherapy

All patients enrolled received neoadjuvant chemotherapy set by the multidisciplinary team (MDT) comprising surgeons, medical oncologists, and radiologists of The Sixth Affiliated Hospital, Sun Yat-sen University. Information on regimen, cycles, and timing was collected.

### 2.3. Surgery

All patients received subsequent curative tumor resection (total or subtotal gastrectomy) with D2 lymphadenectomy. Open or laparoscopic surgery was chosen according to the preference of the surgeon. A thorough examination of the abdominal cavity was routinely performed to determine the status of peritoneal metastasis, while a peritoneal washing cytology test was not routinely conducted. Information on gastrectomy extent, resection approach, surgical findings, postsurgery complications, and pathological findings was collected.

### 2.4. Adjuvant Chemotherapy

Information on completion, regimen, cycles, and timing of adjuvant chemotherapy was collected.

### 2.5. Follow-Up

All follow-up work was conducted by the follow-up office from the gastric cancer database. Information on survival was retrieved.

### 2.6. Data Analysis

The normality of the data was assessed using the Kolmogorov-Smirnov test and normal probability plots. Parameters that were not normally distributed are expressed in the form of medians (upper quartile to lower quartile). Both categorical and continuous variables were analyzed independently with a Cox regression model, and those variables with a *p* value < 0.05 were then enrolled in the multivariable analysis. A stepwise selection method was adopted to develop a regression model with maximum predictive power. A nomogram was then developed upon the established model. The concordance statistic was acquired for the nomogram, and internal validation using the bootstrap method was performed to determine the adjusted concordance statistic. A calibration curve and receiver operating curve (ROC) for the nomogram were generated to show the prediction efficiency of the model. All statistical analyses were performed using SPSS software ver. 22.0 (IBM, Armonk, NY, USA) and R version 3.6.1 software (The R Foundation for Statistical Computing, Vienna, Austria; http://www.r-project.org/).

## 3. Results

A total of 215 patients with a histological diagnosis of adenocarcinoma of the stomach or esophagogastric junction from March 2012 to December 2019 were enrolled in the study. As depicted in Table 1, the majority of patients were male (167/215, 77.7%). The average age of the cohort was 57 ± 11 years old. Tumors were predominantly poorly differentiated (grade 3, 148/215, 68.8%), with radiologically suspicious lymph node metastasis (210/215, 97.7%).

All patients received treatments as depicted in Table 2, including NAC, surgery, and adjuvant chemotherapy. Patients received a median of 4 cycles of NAC. More than half (121/215, 56.3%) of the patients received the mFLOT regimen, and other mainstream regimens included SOX (60/215, 27.9%), FOLFOX (11/215, 5.1%), and XELOX (7/215, 3.3%). Subsequent radical resection followed after an average of 29 ± 10 days. The most common resection approach was laparoscopy. A thorough abdominal exploration was routinely conducted for all patients before resection, and 3.3% (7/215) of patients were confirmed to have occult distal metastasis that was not identified before surgery. Additionally, 11.6% (25/215) of patients received multivisceral resection due to adjacent organ invasion or distal metastasis, but in the end, R0 resection was achieved for most patients (190/215, 88.4%). Major complications (Grade IIIa-V according to the Clavien-Dindo Classification system) included anastomotic leakage (11/215, 5.1%), bleeding (7/215, 3.3%), thoracic effusion (4/215, 1.9%), ileus (2/215, 0.9%), and severe pneumonia (1/215, 0.5%). Nineteen patients were managed by medication therapy or interventions that require no general anesthesia, such as endoscopic hemostasis or ultrasound-guided centesis drainage, and 5 patients were managed by reoperation. One patient eventually died of severe pneumonia after being transmitted to the intensive care unit. After radical resection, the majority of patients received a median of 5 cycles of adjuvant chemotherapy (198/215, 92.1%), mostly FOLFOX (79/215, 36.7%) or its derived regimen SOX (51/215, 23.7%); others included a docetaxel-based regimen (42/215, 19.5%) and oral fluorouracil (26/215, 12.1%).

The pathological findings are depicted in Table 3. Pathological complete response (pCR) was achieved in 13% (28/215) of patients. The average number of examined lymph nodes (exLNs) was 26 ± 13, and half of the patients (117/215, 54.4%) had lymph node metastasis in the final pathological analysis, significantly less than estimated presurgically (cN+: 210/215, 97.7%).

**Figure 2:**
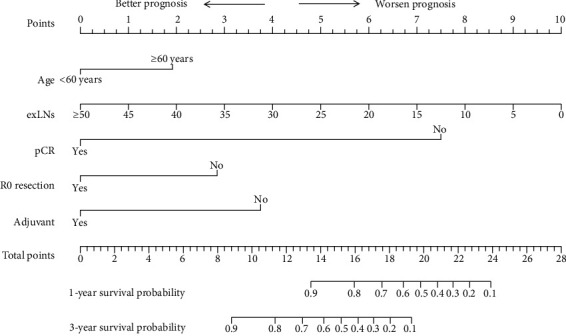
Parameters in Tables 1–3 with a *p* value < 0.05 were included in the Cox regression model to build a nomogram predicting 1- and 3-year survival after neoadjuvant chemotherapy and surgery. exLNs: examined lymph nodes; pCR: pathological complete response; Adjuvant: adjuvant chemotherapy.

**Figure 3:**
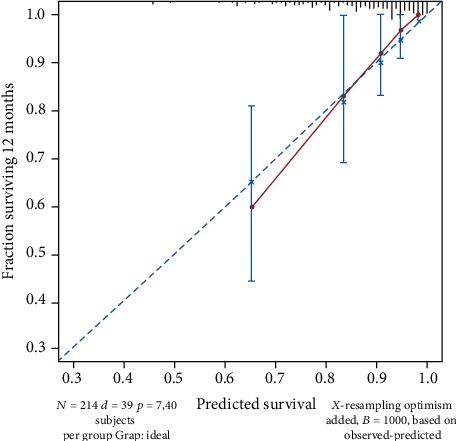
Calibration curve showing a good fitting of the predicted and observed survival.

**Figure 4:**
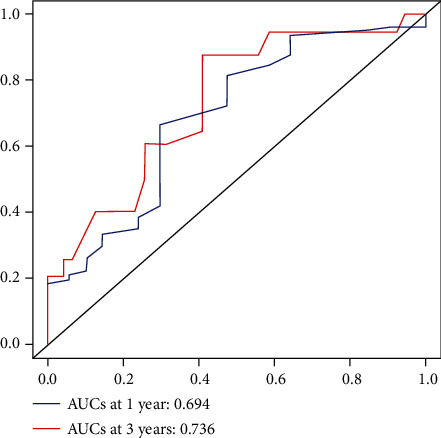
Receiver operating curve (ROC) of the nomogram at 1/3 years.

In the survival analysis, the median follow-up duration of the cohort was 12 (5-21) months, with 39 cases of tumor-related death observed during this period. The overall 1-year and 3-year survival rates were 85.8% and 55.6%, respectively. Tables 1–3 show the hazard ratios, 95% confidence intervals, and the respective *p* value of each parameter in univariable survival analysis. Parameters that had a significant impact on survival (*p* < 0.05) were age, R0 resection, vascular tumor embolus, nerve invasion, pCR, number of exLNs, and adjuvant chemotherapy. These parameters were enrolled in multivariable analysis and selected by the stepwise procedure to build a model with the strongest predictive power (Akaike information criterion statistic = 320). In the final established Cox proportional hazards model, younger age (<60 years old), increased exLNs, successful R0 resection, pCR, and receiving adjuvant chemotherapy were predictors for prolonged survival.  Table 4 summarizes the hazard ratio and 95% confidence interval of each predictor. A nomogram predicting survival after NAC and radical resection was constructed according to the established model, as shown in Figure 2. The apparent C-index of the nomogram was 0.785, indicating a satisfactory efficiency in predicting survival. Calibration curves demonstrated a good fitting between predicted and actual observations of survival, indicating an ideal statistical performance for predicting survival, as shown in Figure 3. The areas under the curve (AUC) of receiver operating curve (ROC) at 1/3 years were 0.694/0.736, respectively, showing a good discriminative power of the model (Figure 4).

## 4. Discussion

In our study, we found that younger age (<60 years old), increased exLNs, successful R0 resection, pCR, and receiving adjuvant chemotherapy were positive predictors of survival. A nomogram was established according to the weights of these predictors in the model, with exLNs and pCR having the largest impact on survival. All factors included in the model are easily available in clinical practice, and internal validation showed consistent and stable predictive power, making it a practical tool for clinical reference.

There have been many studies trying to identify survival predictors for AGC patients who received NAC and radical surgery, but most of these studies only show which factors may be related to survival without specifying the weights and impact of these factors; a panoramic model that describes the survival of this patient subgroup is lacking. Nomogram is a prediction model based on a Cox regression model consisted of axes and a scoring system, each axis represents an independent survival predictor, and the corresponding score on the axis represents the impact of the predictor. It gives an easily perceptible visualization of the survival of a specific disease. To our knowledge, this is the first nomogram integrating clinical parameters from different treatment phases for AGC patients after NAC and surgery. Most of the parameters enrolled in the final model emphasize the importance of complete elimination of the tumor. First, for the number of exLNs, according to the 8^th^ edition of the AJCC staging manual, the retrieval of at least 16 lymph nodes is the minimal requirement for lymph node dissection [20], but there is growing evidence that increasing the number of harvested lymph nodes significantly improves survival [21–28]. Increased exLNs indicate a more extended lymph node dissection and a more thorough clearance of the tumor. As shown in our model, exLNs are positively relative to the survival, with the longest axis, indicating the great importance of complete tumor clearance. Second, pCR is another significant positive predictor of survival in our model, with the second-longest axis (non-pCR vs. pCR, HR: 9.06, 95% CI: 1.22–67.4). It has been well established that pCR is closely related to prolonged survival after NAC and radical resection [29–31]. Previous data showed that those with pCR following NAC could achieve a 5-year survival rate of up to 89%, which is very favorable among AGC patients [20]. Thus, NAC is highly recommended for patients with the potential of achieving pCR. However, pCR is uncommon, and our data showed that only 13% of patients achieve pCR, similar to previous reports (8.4–17.4%) [32–35]. A few studies have been devoted to identifying the predictive factors of pCR; possible positive factors include good differentiation of the tumor cells, higher carcinoma embryonic antigen levels and lymphocyte ratios, and lower monocyte counts [36, 37]. Patients with these clinical features tend to benefit more from NAC. Third, evidence in recent years has shown that R0 resection is potentially beneficial for AGC patients, even those with limited metastasis or at the cost of multivisceral resection [38, 39], and the target of NAC is also to achieve R0 resection with minimal morbidity and mortality and eventually prolonged survival and better quality of life [40]. In our study, limited occult metastasis was discovered in 3.3% (7/215) of patients, and multivisceral resection was performed in 11.6% (25/215) of patients due to adjacent organ invasion or limited distal metastasis, but their survival did not seem to significantly deteriorate as long as R0 resection could be achieved, reemphasizing the importance of complete resection of the tumor. Last, the importance of adjuvant chemotherapy for AGC after radical resection has been well recognized for years [41]. Adjuvant chemotherapy helps to eliminate residual tumor cells and increase the chance for disease-free survival. As shown in our model, adjuvant chemotherapy still had a positive impact on survival after NAC and radical surgery; therefore, it should be recommended as an essential part of the treatment modality.

There are a few limitations to our study. First, it is noticeable that the ypT stage and ypN stage are not included in the model; this is because the distribution of patients across different stages was very imbalanced, as shown in Table 3. This weakens the discrimination power of the survival data. Additionally, pCR is closely related to ypT and ypN stages, and including these codependent factors in the same model would have resulted in an interaction effect that would have weakened the predictive power of the model. Second, the model was validated internally using the bootstrap method, lacking external validation in an independent cohort from a different institution, but the same validation approach has been used by many previous studies and has been proven to be efficient [42, 43]. Third, the robustness of the nomogram is limited by the sample size.

## 5. Conclusion

A nomogram predicting the survival of AGC patients after NAC and surgery was established. To improve survival, physicians should aim at achieving pCR and R0 resection and harvesting more lymph nodes. Adjuvant chemotherapy is also highly recommended after radical resection.

## Figures and Tables

**Table 1:** Patient characteristics, hazard ratios, and *p* values from univariable survival analysis (*n* = 215).

	*n* (%)	Hazard ratio (95% CI)	*p* value
Age (years)			
*<60*	113 (52.6%)	Reference	—
*≥60*	102 (47.4%)	2.34 (1.23-4.46)	0.01
Sex			
*Male*	167 (77.7%)	Reference	—
*Female*	48 (22.3%)	1.49 (0.75-2.94)	0.26
BMI (kg/m^2^)	21.8 ± 2.9	0.99 (0.89-1.11)	0.87
*0-24*	173 (80.5%)	Reference	—
*>24*	42 (19.5%)	0.73 (0.31-1.75)	0.48
Location			
*Upper*	85 (39.5%)	Reference	—
*Middle*	38 (17.7%)	0.96 (0.40-2.31)	0.93
*Lower*	85 (39.5%)	0.73 (0.36-1.47)	0.38
*Whole*	7 (3.3%)	0.005 (0-10+)	0.72
Tumor grade			
*G3*	151 (70.2%)	Reference	—
*G2*	56 (26%)	0.78 (0.38-1.62)	0.51
*G1*	8 (3.7%)	0.006 (0-10+)	0.65
Clinical T stage			
*cT3*	109 (50.7%)	Reference	—
*cT4*	106 (49.3%)	1.61 (0.83-3.16)	0.16
Clinical N staging			
*cN0*	5 (2.3%)	Reference	—
*cN+*	210 (97.7%)	1.61 (0.21-12.21)	0.645

**Table 2:** Treatment information on chemotherapy and surgery, hazard ratios, and *p* values from univariable survival analysis (*n* = 215).

	*n* (%)	Hazard ratio (95% CI)	*p* value
Neoadjuvant regimen			
*mFLOT*	121 (56.3%)	Reference	
*SOX, XELOX or FOLOFX*	78 (36.3%)	0.45 (0.19-1.05)	0.07
*Other*	16 (7.4%)	0.39 (0.15-1.02)	0.05
Cycles received	4 (4-4)	1.08 (0.88-1.34)	0.45
Time gap between NAC and surgery (days)	29 ± 10	1.03 (1-1.05)	0.07
Resection extend			
*Total gastrectomy*	129 (60%)	Reference	—
*Subtotal gastrectomy*	86 (40%)	0.76 (0.39-1.47)	0.42
Laparoscopic surgery			
*Yes*	167 (77.7%)	Reference	—
*No*	48 (22.3%)	1.45 (0.76-2.78)	0.26
Metastasis found during surgery			
*Yes*	7 (3.3%)	Reference	—
*No*	208 (96.7%)	0.83 (0.11-6.08)	0.83
Multivisceral resection			
*Yes*	25 (11.6%)	Reference	—
*No*	190 (88.4%)	0.64 (0.26-1.54)	0.32
R0 resection			
*Yes*	190 (88.4%)	Reference	—
*No*	25 (11.6%)	0.40 (0.20-0.83)	0.01
Major complication^∗^			
*No*	191 (88.8%)	Reference	—
*IIIa*	24 (11.2%)	0.67 (0.21-2.18)	0.51
Reoperation within 30 days			
*Yes*	5 (2.3%)	Reference	—
*No*	210 (97.7%)	0.72 (0.19-10+)	0.75
Adjuvant chemotherapy			
*No*	17 (7.9%)	Reference	—
*Yes*	198 (92.1%)	0.27 (0.12-0.62)	<0.01
Cycles received	5 (3-5)	0.88 (0.78-1)	0.05
Time between surgery and adjuvant chemotherapy (days)	36 ± 19	1.03 (1-1.05)	0.07

mFLOT: docetaxel 50~60 mg/m^2^ + oxaliplatin 85 mg/m^2^ + fluorouracil 2800 mg/m^2^ iv over 48 hours, every 2 weeks; SOX: oxaliplatin 130 mg/m^2^ iv + tegafur/gimeracil/oteracil potassium capsule 40~60 mg bid D1-D14 every 3 weeks; XELOX: oxaliplatin 130 mg/m^2^ + capecitabine 1000 mg/m^2^ bid D1-D14 every 3 weeks; FOLFOX: oxaliplatin 85 mg/m^2^ + fluorouracil 2800 mg/m^2^ civ over 48 hours every 2 weeks. The dosage of the regimens above might be modified according to the preference of the oncologist. NAC: neoadjuvant chemotherapy. ^∗^Major complication is defined according to the Clavien-Dindo Classification system (Grade III and above): Grade III, complications requiring surgical, endoscopic, or radiological intervention (IIIa: no general anesthesia required; IIIb: general anesthesia required); Grade IV, life-threatening complications requiring IC/ICU management; Grade V, death.

**Table 3:** Pathological findings, hazard ratios, and *p* values from univariable survival analysis (*n* = 215).

	*n* (%)	Hazard ratio (95% CI)	*p* value
Pathological complete response			
*No*	187 (87%)	Reference	—
*Yes*	28 (13%)	0.1 (0.01-0.77)	0.03
ypT stage			
*T0*	28 (13%)	0.09 (0.01-0.66)	0.02
*T1*	26 (12.1%)	0.46 (0.14-1.52)	0.20
*T2*	21 (9.8%)	0.43 (0.13-1.44)	0.17
*T3*	136 (63.3%)	Reference	—
*T4*	4 (1.9%)	1.33 (0.3-5.92)	0.70
Harvested lymph nodes	26 ± 13	0.95 (0.92-0.98)	<0.01
ypN stage			
*N0*	98 (45.6%)	Reference	—
*N1*	51 (23.7%)	1.83 (0.81-4.14)	0.15
*N2*	33 (15.3%)	2.0 (0.74-5.43)	0.17
*N3a*	26 (12.1%)	3.64 (1.42-9.35)	<0.01
*N3b*	7 (3.3%)	4.89 (1.36-17.6)	0.02
Vascular tumor embolus			
*No*	175 (81.4%)	Reference	—
*Yes*	40 (18.6%)	2.31 (1.12-4.78)	0.02
Nerve invasion			
*No*	143 (66.5%)	Reference	—
*Yes*	72 (33.5%)	2.03 (1.07-3.84)	0.03
HER2			
*(-)*	117 (54.4%)	Reference	—
*(+)~(++)*	53 (24.7%)	1.22 (0.6-2.46)	0.58
*(+++)*	4 (1.9%)	5.57 (1.63-19)	<0.01
*Data missing*	41 (19.1%)		

**Table 4:** Hazard ratio, 95% confidence interval, and *p* value of each predictor in the Cox regression model (*n* = 215).

	Hazard ratio	95% CI	*p*-value
Age			
*≥60 years*	1.76	0.9–3.43	0.09
*<60 years*	Reference		
PCR (No vs Yes)			
*No*	9.06	1.22–67.4	0.03
*Yes*	Reference		
R0 resection (No vs Yes)			
*No*	2.31	1.11–4.83	0.03
*Yes*	Reference		
Adjuvant chemotherapy (Yes vs No)			
*Yes*	0.33	0.14–0.79	0.01
*No*	Reference		
exLNs (Increase per 1 node)	0.94	0.91–0.98	<0.01

## Data Availability

The datasets used and analyzed during the current study are unavailable due to the privacy policy of our institution.
